# Factors Influencing the Acceptance and Adoption of Mobile Health Apps by Physicians During the COVID-19 Pandemic: Systematic Review

**DOI:** 10.2196/50419

**Published:** 2023-11-08

**Authors:** Sultan Alsahli, Su-yin Hor, Mary Lam

**Affiliations:** 1 School of Public Health Faculty of Health University of Technology Sydney Sydney Australia; 2 Department of Health Information Technology and Management College of Public Health and Health Informatics Umm Al-Qura University Makkah Saudi Arabia; 3 Department of Health and Biomedical Sciences STEM College RMIT University Melbourne Australia

**Keywords:** mobile health, mHealth, mobile app, adoption, acceptance, barrier, attitude, physician, doctor, practitioner, mobile phone

## Abstract

**Background:**

During the COVID-19 pandemic, the provision of and access to health care have been uniquely challenging, particularly during lockdowns or when dealing with COVID-19 cases. Health care professionals have had to provide patients with the necessary health care. However, delivering health care services while reducing face-to-face interaction puts an immense strain on health systems that are already overburdened. Against this backdrop, it is now more critical than ever to ensure the accessibility of health care services. Such access has been made increasingly available through mobile health (mHealth) apps. These apps have the potential to significantly improve health care outcomes and expectations and address some of the challenges confronting health care systems worldwide. Despite the advantages of mHealth, its acceptance and adoption remain low. Hence, health care organizations must consider the perceptions and opinions of physicians if the technology is to be successfully implemented.

**Objective:**

The objective of this systematic review was to explore and synthesize the scientific literature on the factors influencing the acceptance and adoption of mHealth among physicians during the COVID-19 pandemic.

**Methods:**

A systematic review of the studies published between March 2020 and December 2022 was conducted using the MEDLINE, Scopus, Embase, and ProQuest databases. The database search yielded an initial sample of 455 potential publications for analysis, of which 9 (2%) met the inclusion criteria. The methodology of this review was based on PRISMA (Preferred Reporting Items for Systematic Reviews and Meta-Analyses).

**Results:**

The factors influencing mHealth acceptance and adoption by physicians were divided into perceived barriers and perceived facilitators, which were further grouped into the following 3 major thematic categories: technological, individual, and organizational barriers and facilitators, respectively. The technological barriers were accessibility, technical issues, usefulness, and data management; individual barriers were perceived patient barriers, time and workload pressure, technical literacy, knowledge of mHealth, and peer support; and organizational barriers were financial factors, management support and engagement, data security, telemonitoring policy, and collaboration. The technological facilitators of uptake were technical factors, clinical usefulness, and data management; individual facilitators were patient-related care, intrinsic motivation, collaboration, and data sharing (individual); and organizational facilitators were workflow-related determinants, organizational financial support, recommendation of mHealth services, and evidence-based guidelines.

**Conclusions:**

This review summarized the evidence on the factors influencing mHealth acceptance and adoption by physicians during the COVID-19 pandemic. The main findings highlighted the importance of addressing organizational readiness to support physicians with adequate resources, shifting the focus from technological to patient-centered factors, and the seamless integration of mHealth into routine practice during and beyond the pandemic.

**Trial Registration:**

PROSPERO CRD42022356125; https://tinyurl.com/2mmhn5yu

## Introduction

### Background

On March 11, 2020, the World Health Organization (WHO) declared the outbreak of the COVID-19 pandemic [1], a crisis that has put pressure on health care systems around the world [2,3], with multiple waves of infections and deaths [4,5]. A recent report by the WHO stated that there have been 757,264,511 confirmed cases of COVID-19, of which 6,850,594 (0.9%) have been fatalities [6].

During this period, the provision of and access to health care have been uniquely challenging [3,7,8], particularly during lockdowns or when dealing with COVID-19 cases. Health care professionals have had to provide patients with the necessary health care. However, delivering health care services while reducing face-to-face interaction puts an immense strain on health systems that are already overburdened [9]. Against this backdrop, it is now more critical than ever to ensure the accessibility of health care services. Such access has been made increasingly available through mobile health (mHealth) apps, given the advancements in information and communication technology. These apps have the potential to significantly improve health care outcomes and expectations and address some of the challenges confronting health care systems worldwide [10-14].

mHealth falls under the broader umbrella of eHealth, which encompasses the use of electronic technologies and digital communication to enhance health care delivery [15-17]. However, mHealth technologies differ from conventional eHealth technologies in that they are specifically designed for use on mobile devices, and as such, mHealth apps do not rely solely on computers and wired internet connections, which makes them more accessible [18]. In addition, mHealth extends beyond medical consultations (more commonly known as telemedicine), offering features such as symptom tracking, mental health support, fitness tracking, medication reminders, personalized support, and access to health-related information [18-21]. Using mHealth is a popular strategy because it is user driven, readily available, and often reasonably priced [22].

The WHO [23] acknowledged that there is no widely accepted definition of mHealth, but it could be understood as the practice of using mobile devices for health care. More specifically, it refers to the capability to use mobile devices to collect health care–relevant data from patients in real time and use such information to monitor, diagnose, and treat patients [24]. It has the potential to benefit both health care professionals and patients during the COVID-19 pandemic [14,25,26]. For instance, it can improve the delivery of health care services, reduce health care professional and patient exposure to infectious diseases, and minimize patient demand for facilities [27,28]. In addition, mHealth apps use location data and proximity alerts to notify users if they were in close contact with someone who later tested positive for COVID-19 [29,30]. These timely alerts empower people to self-isolate, get tested, and inform their health care providers, helping break the chain of transmission [29,31]. It also offers opportunities for health care professionals to remotely consult and share data with their colleagues [32,33]. Furthermore, mHealth not only enables patients to receive remote consultation but also improves their adherence to medication and delivers disease education [20,25,33,34].

Despite the above-mentioned advantages, the acceptance and adoption of mHealth remain low [35-38]. The factors that influence technology acceptance and adoption are likely to vary across target users [39,40]. Physicians, for example, can stimulate changes in the health care sector and play a critical role in mHealth acceptance and adoption, depending on whether they themselves embrace this new technology. As explained by Cajita et al [41], patients are willing to accept and adopt mHealth when their physicians recommend it. Hence, health care organizations must consider the perceptions and opinions of physicians if the technology is to be successfully implemented [42].

### Objectives

Before the COVID-19 pandemic, the acceptance and adoption of technology for work duties were a matter of personal or organizational preference [43]. This orientation was changed by the crisis, which compelled technology use in work environments, thereby accelerating the process of digitization in all sectors, including health care. As previously stated, physicians have been forced to provide health care services remotely [44], and they have accepted and adopted mHealth because of physical distancing restrictions. This situation may affect their continued use of the technology, which is one of the success factors for acceptance and adoption [38]. However, Keuper et al [44] found in their study that only a few physicians intend to continue offering remote health care services in the future. A possibility is that the COVID-19 pandemic has changed the behavioral intentions and perceptions of people regarding digital transformation [45,46]. Thus, the factors influencing technology acceptance and adoption have also likely changed [47], or new factors might have emerged. Shedding light on these factors can facilitate the acceptance and adoption of mHealth and help health care professionals provide services during the COVID-19 pandemic and other similar crises in the future.

Although previous reviews have analyzed mHealth acceptance and adoption by physicians [42,48,49], to the best of our knowledge, none of these reviews have focused on this topic in the context of the COVID-19 pandemic. This systematic review intended to fill this void. This review can benefit policy makers and mHealth providers by presenting an updated and thorough assessment of important issues that affect mHealth acceptance and adoption among physicians. This review can also help them design a strategy for promoting mHealth acceptance and adoption and derive potential benefits from this technology. Finally, this review provides opportunities for follow-up research by identifying potential gaps in mHealth acceptance and adoption.

## Methods

### Overview

The methodology of this review was based on the PRISMA (Preferred Reporting Items for Systematic Reviews and Meta-Analyses) [50], which provides guidelines for a reliable and rigorous literature review (Multimedia Appendix 1). The review protocol was registered and published in advance with PROSPERO (CRD42022356125). The review focused on quantitative, qualitative, and mixed method studies to identify the factors that influenced the acceptance and adoption of mHealth among physicians during the COVID-19 pandemic.

### Search Strategy

MEDLINE (Ovid), Scopus (Elsevier), Embase (Ovid), and ProQuest databases were searched for studies published in the English language. As the aim of this review was to explore mHealth acceptance and adoption factors during the pandemic, the time frame selected was from 2020 to 2022. The search strategy was established based on the population, intervention, comparator, and outcome (PICO) framework [51]. Specifically, we searched for studies revolving around physicians (population); the use of mHealth apps, including smartphones, portable digital devices, and tablets (intervention); and mHealth acceptance and adoption (outcome). Comparators were not relevant to this review.

Initially, combinations of Medical Subject Headings (MeSH) terms, keywords, and terminologies were used with reference to the following 3 categories: “mHealth,” “acceptance or adoption,” and “physician” (Multimedia Appendix 2). The more specific search terms used were as follows: “mobile health,” “mHealth,” “mHealth,” or “mobile app”; “adoption,” “acceptance,” “barrier,” or “attitude”; and “physician,” “doctor,” or “practitioner.”

### Study Selection

We used Covidence (Veritas Health Innovation, Ltd), a web-based collaboration software platform, to support the screening of the identified studies, all of which were uploaded onto the platform. A 2-step screening procedure was conducted to evaluate the relevance of the studies. In the first step, the titles and abstracts of the studies were screened independently by 2 reviewers (SA and SH). Any disagreements between the reviewers at the first step were discussed until a consensus was reached, or a third reviewer assisted in resolving the disagreement. In the second step, the studies that met the inclusion criteria were subjected to full-text screening carried out independently by 2 reviewers (SA and ML). Any disagreements at this point were resolved through discussion, or a third reviewer (SH) aided in the resolution.

### Inclusion and Exclusion Criteria

The inclusion criteria were studies that (1) focused on the acceptance and adoption of mHealth primarily by physicians, (2) addressed factors influencing acceptance and adoption, (3) were peer reviewed, and (4) were published in English. The exclusion criteria were studies that (1) examined other health care technologies, such as electronic health records and electronic medical records; (2) focused solely on participants other than physicians (ie, patients, nurses, and midwives); and (3) collected data before the COVID-19 pandemic.

### Quality Assessment

The studies included in the final data synthesis were assessed for methodological quality using the Quality Assessment with Diverse Studies (QuADS) criteria [52]. The QuADS is a 13-criteria tool developed to evaluate the quality of different designs, including quantitative, qualitative, and mixed methods research. For each criterion, a study can derive a score ranging from 0 (no mention at all) to 3 (full details), with the maximum possible score being 39. A QuADS score was calculated for each study, after which the item scores were summed and divided by the maximum possible score to obtain an overall quality assessment for each study. Studies with scores lower than 50%, ranging from 50% to 70%, and greater than 70% were classified as being of low, moderate, and high methodological quality, respectively [53]. Two authors (SA and SH) independently assessed the studies, and disagreements were resolved through discussion (Multimedia Appendix 3 [28,54-61]).

### Data Extraction and Synthesis

Given the heterogeneous factors identified in the included studies, conducting a meta-analysis synthesis was not possible. Instead, the results on factors influencing the acceptance and adoption of mHealth among physicians were narratively synthesized. The selected studies were subjected to data extraction, with their titles, abstracts, and full texts screened, after which the required information was obtained using a predefined data extraction form. This form included the following details: authors, year of publication, location, study design, sample size, targeted population, theoretical framework, and influencing factors. To ensure the validity of these details, 2 reviewers (SA and ML) independently recorded them. Differences or disagreements were resolved through discussion.

## Results

### Overview

**Figure 1:**
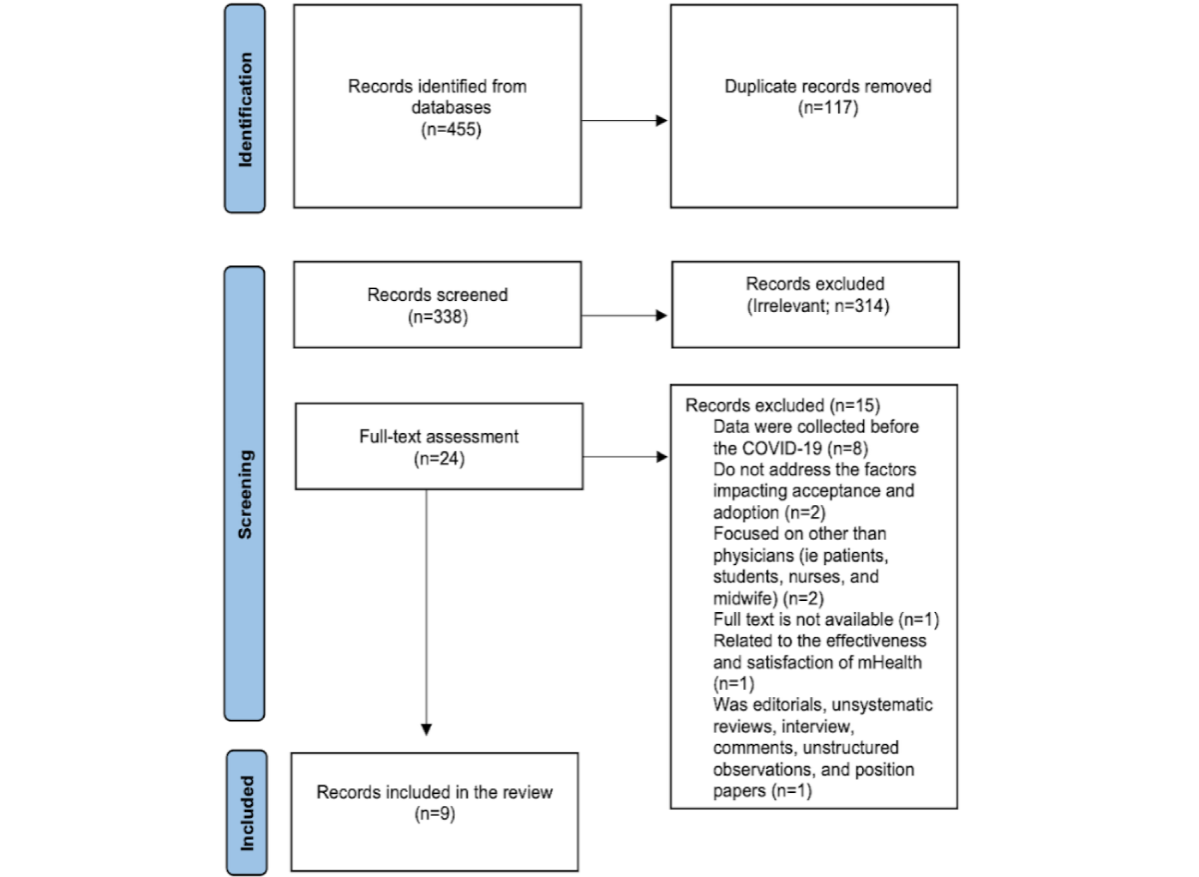
PRISMA (Preferred Reporting Items for Systematic Reviews and Meta-Analyses). mHealth: mobile health.

The database search yielded an initial sample of 455 potential publications for analysis. Of these 455 publications, 117 (25.7%) duplicates were eliminated. The titles and abstracts of the remaining 338 (85.3%) publications were reviewed, resulting in 314 (92.9%) publications being discarded at this stage for failing to meet the inclusion criteria. This remaining 24 (7.1%) publications underwent full-text review, of which 15 (62%) were eliminated because they did not meet the inclusion criteria (Figure 1). The final sample consisted of 9 published papers, whose key features are highlighted in Table 1.

**Table 1:** Characteristics of the included studies (N=9).

Study	Country	Study Design	Participants (physicians), n	Targeted population	Specialty	Theoretical framework	Assessment tool	QuADS^a^ score (%)
Aquino et al [54]	Canada	Qualitative	5	Clinicians and patients	Obstetricians	NR^b^	Interviews	59
Artanian et al [28]	Canada	Qualitatibe	5	Clinicians and patients	Cardiologists	Chaudoir multilevel framework	Interviews	87
Bhatt and Chakraborty [55]	India	Quantitative	316	Physicians	Multiple specialties	UTAUT^c^	Questionnaire	51
Dahlhausen et al [56]	Germany	Mixed methods	1295	Physicians	Multiple specialties	NR	Interviews and questionnaire	79
Fleddermann et al [57]	United States	Qualitative	13	Physicians	Multiple specialties	NR	Interviews	69
Jackson et al [58]	United States	Qualitative	29	Clinicians (physicians, nurses, diabetes educators, and dietitians)	Obstetricians	Stakeholder co-design framework	Focus groups and interviews	77
Li et al [59]	Australia	Qualitative	13	Clinicians and patients	Obstetricians	NR	Interviews	59
Mansour [60]	Egypt	Quantitative	203	Physicians	Multiple specialties	NR	Questionnaire	51
Wu et al [61]	China	Quantitative	393	Physicians	Multiple specialties	UTAUT	Questionnaire	72

^a^QuADS: Quality Assessment with Diverse Studies.

^b^NR: not reported.

^c^UTAUT: Unified Theory of Acceptance and Use of Technology.

### Characteristics of the Included Studies

As shown in Table 1, of the 9 included studies, 2 (22%) each were conducted in the United States [57,58] and Canada [28,54], whereas 1 (11%) each was conducted in India [55], Australia [59], China [61], Egypt [60], and Germany [56]. A total of 5 (56%) studies focused on physicians [55-57,60,61], and 2 (22%) studies included patients as well [28,59]. Moreover, 1 (11%) study included practicing nurses in addition to physicians and patients [54], whereas another (11%) involved physicians, nurses, diabetes educators, dietitians, and lactation counselors [58]. From the perspective of specialization, most studies (5/9, 56%) involved physicians with multiple specialties [55-57,60,61], whereas other studies (4/9, 44%) involved cardiologists and obstetricians [28,54,58,59]. More than half (5/9, 56%) of the studies did not mention the use of a theoretical framework. A total of 2 (22%) studies used the Unified Theory of Acceptance and Use of Technology [55,61], 1 (11%) adopted a stakeholder co-design framework [58], and another used the Chaudoir multilevel framework [28]. Most studies (5/9, 56%) followed a qualitative approach that entailed conducting semistructured interviews and focus group discussions [28,54,57-59]. Overall, 3 (33%) studies adopted a quantitative approach entailing questionnaire administration [55,60,61], and only 1 (11%) used a mixed methods approach, in which questionnaires were administered and semistructured interviews were conducted [56].

### Quality Assessment

As mentioned earlier, the studies were assessed using the QuADS tool to evaluate quality and risk of bias [52]. The methodological quality of the examined studies ranged from 51% to 87%. Overall, 4 (44%) studies had high-quality methodologies (scores of 72% to 87%), 5 (56%) studies had moderate-quality methodologies (scores ranging from 51% to 69%), and no study had low scores.

### Factors Affecting Physicians’ Acceptance and Adoption of mHealth Technologies

#### Perceived Barriers

##### Overview

**Figure 2:**
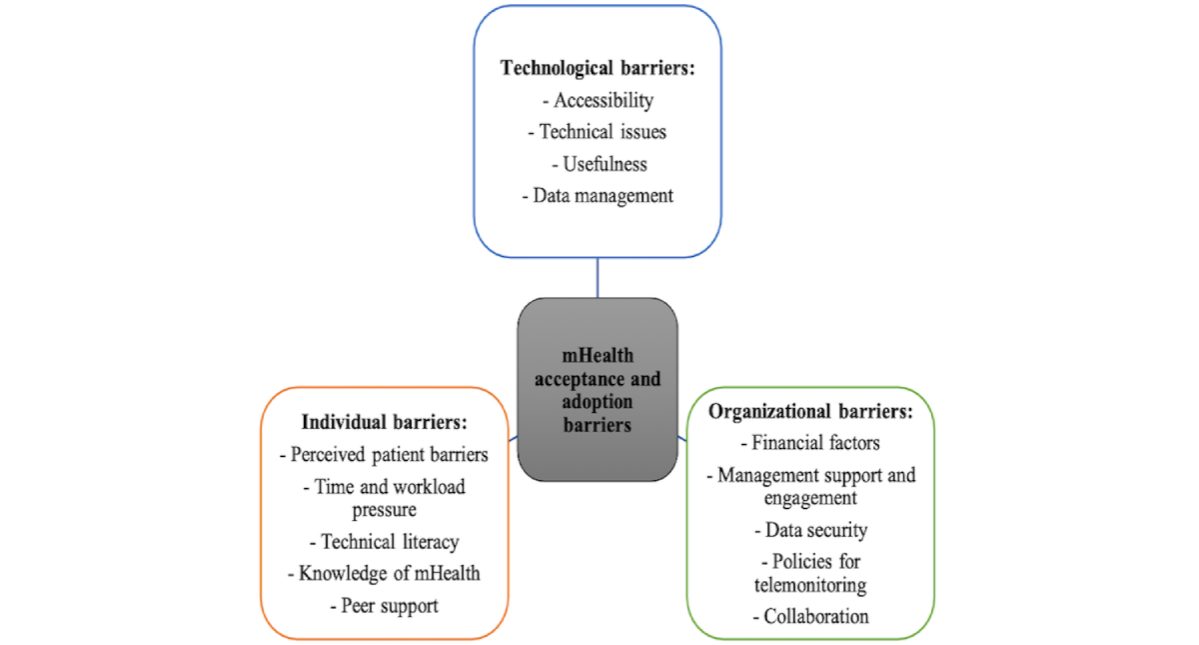
Themes of barriers to mobile health (mHealth) acceptance and adoption by physicians.

All but 1 (11%) [61] of the 9 reviewed papers reported on perceived barriers to the acceptance and adoption of mHealth technologies by physicians. These barriers are summarized in Table 2. The literature is characterized by inconsistency in the use of theoretical frameworks to categorize barriers, and no single framework captures all relevant factors without some form of extension. Therefore, in this review, perceived barriers were grouped based on common themes and mapped into the following 3 major thematic categories: technological, individual, and organizational barriers (Figure 2).

**Table 2:** Barriers to the acceptance and adoption of mobile health (mHealth) technologies among physicians.

Study	Technological barriers	Individual barriers	Organizational barriers
Aquino et al [54]	Lack of availability of telemonitoring systems for patients at a high risk for preeclampsia Clinical utility: additional value in care management	Increased clinician workload	Lack of health system policies: limited guidelines for the telemonitoring of patients at a high risk for preeclampsia Lack of access to appropriate resources (eg, validated BP^a^ cuffs) Financial cost (eg, cost of home BP monitor for patients)
Artanian et al [28]	Lack of preparedness to implement telemonitoring: uncertainty regarding the functionality, operationalization, and integration of technology	Patient preference for face-to-face contact Patient acceptance of long-term technology use	Lack of resources for supporting telemonitoring intervention: in the absence of a dedicated coordinator, time consuming for clinicians Financial and economic factors: costs associated with resources for sustaining telemonitoring (eg, additional staff) Physician remuneration: lack of compensation for services
Bhatt and Chakraborty [55]	NR^b^	Limited confidence (technology anxiety) Lack of skill set for using mHealth services	NR
Dahlhausen et al [56]	Technical concerns: training needs, technical integration issues, and lack of technical support Clinical utility: uncertainties about benefits and insufficient medical evidence Low availability of technology	Increased workload Lack of awareness Perceived low competence due to insufficient knowledge about differentiating mHealth platforms Medicolegal concerns about potential liabilities for mistreatment	Data protection and security Financial factor: lack of reimbursement for mHealth-related medical services Limitations of infrastructures: workflow-related issues (eg, workflow adjustments and training needs)
Fleddermann et al [57]	Lack of adequate access to technology (among patients) Challenges in navigating the technology Competition from other similar apps Lack of relatable content	Lack of time Competing priorities Perceived lack of patient motivation (resistance to change) Lack of peer support during internet-based treatment Lack of in-person interaction for guiding patient use of mHealth	Uncertainty regarding privacy and confidentiality Limited organizational support and engagement Limitations of infrastructures and workflows Pandemic impact: disruption to the provision of services and challenges in shifting to hybrid care delivery and retaining patients
Jackson et al [58]	Lack of evidence-based mHealth resources Reliability of internet resources Concern over ease of use and operationalization Lack of credibility	Limited familiarity, awareness, and knowledge of mHealth availability and utility Low patient engagement in the long term	Formal organizational structure: reliance on provider knowledge networks
Li et al [59]	Accuracy of devices and uncertainty about technology reliability Challenges related to integration with other health record systems Clinical utility or usefulness: lack of evidence on the effectiveness of mHealth monitoring in pregnancy	Pregnant women needing training to measure BP correctly Difficulty with the sustainability of and compliance with the collection of data on pregnant women, especially due to cultural and linguistic barriers Extra workload due to the review of monitoring data Skill set required to accurately analyze the data	Limited communication among clinicians from multiple disciplines: multidisciplinary approach or communication needed to consider pregnancy symptoms, risk factors, test findings, and data about babies Concerns about patient data privacy Limitation of resources for supporting mHealth Financial cost of technology (especially among patients from low socioeconomic backgrounds)
Mansour [60]	Lack of training on using mHealth technologies Lack of appropriate and relevant content Failure of mobile network connection Potential for the misuse of collected information	Lack of time for using technology Lack of technical skills Lack of interest in, knowledge about, or awareness of the benefits of mHealth technologies Lack of language skills Communication barriers: demographic characteristics of patients (age, education, and gender)	Financial cost of technology implementation Concerns about personal data privacy and security
Wu et al [61]	NR	NR	NR

^a^BP: blood pressure.

^b^NR: not reported.

##### Technological Barriers

The technological barriers to acceptance and adoption were further classified into the following 4 key subthemes identified from 8 (89%) of the 9 examined studies: accessibility, technical issues, usefulness, and data management. Technical issues were the most frequently reported barriers, including functionality (eg, concern over ease of use and operationalization) [28,57,58] and technical support (eg, technical issues in daily operations) [56]. Features related to usefulness, such as the clinical utility, added value, and evidence-based effectiveness of mHealth in care management (eg, lack of or insufficient evidence of benefit for patients), were other significant impediments to the use of mHealth technologies [54,56,58,59]. Concerns related to data management, including integration issues (eg, challenges with integration into clinical health records and poor integration or compatibility with existing practice software and tools) [28,56,59], were also raised. Lack of access [54,56,57], reliability [58,59], and limited connectivity (eg, concern about weak or failure of mobile network connectivity) [60] were cited by the rest of the studies.

##### Individual Barriers

Individual intrinsic (eg, confidence) and extrinsic (eg, technical competence) barriers emerged from the 8 (89%) of the 9 explored studies and were categorized into the following 5 key subthemes: perceived patient barriers, time and workload pressure, technical literacy, knowledge of mHealth, and peer support. Patient-related factors were the most prominently cited individual barriers, with patient acceptance or motivation (eg, perceived lack of patient motivation due to resistance to change) and sustained compliance with long-term technology use (eg, difficulty with the sustainability of and compliance with the collection of data on patients, especially due to cultural and linguistic barriers) being central concerns [28,57-60]. Time pressure and extra workload (eg, the additional work required for physicians to monitor patient data) [54,56,57,59,60] were reported as impediments to mHealth use by health care professionals. Other barriers mentioned were limited technical skills and confidence (eg, lack of language skills and technology anxiety) [55,57-60], the lack of knowledge about differentiating between mHealth platforms and awareness of mHealth benefits [56,58,60], and the lack of peer support [57].

##### Organizational Barriers

Organizational barriers were divided into 5 central subthemes: financial factors, management support and engagement, data security, technology policy, and collaboration. The most commonly reported barrier at the organizational level was financial factors, including the cost of mHealth apps and reimbursement issues. These issues involved costs associated with mHealth implementation (eg, the cost of devices) for both physicians [60] and patients [54,59], especially for those with low socioeconomic status [59], and the lack of or insufficient reimbursement for mHealth-related medical services (eg, responding to follow-up questions from patients) [56]. Other central barriers included the need for organizational engagement, lack of human resource support (eg, hiring a dedicated mHealth coordinator to reduce the workload of clinicians), lack of infrastructure [28,54,56-59], and lack of training [60]. The rest of the hindrances to mHealth uptake were the lack of policies related to data security (eg, uncertainty about the privacy and security of personal health data) [56,57,59,60], lack of evidence-based telemonitoring guidelines [54], and lack of communication among health care providers [59].

#### Perceived Facilitators

##### Overview

**Figure 3:**
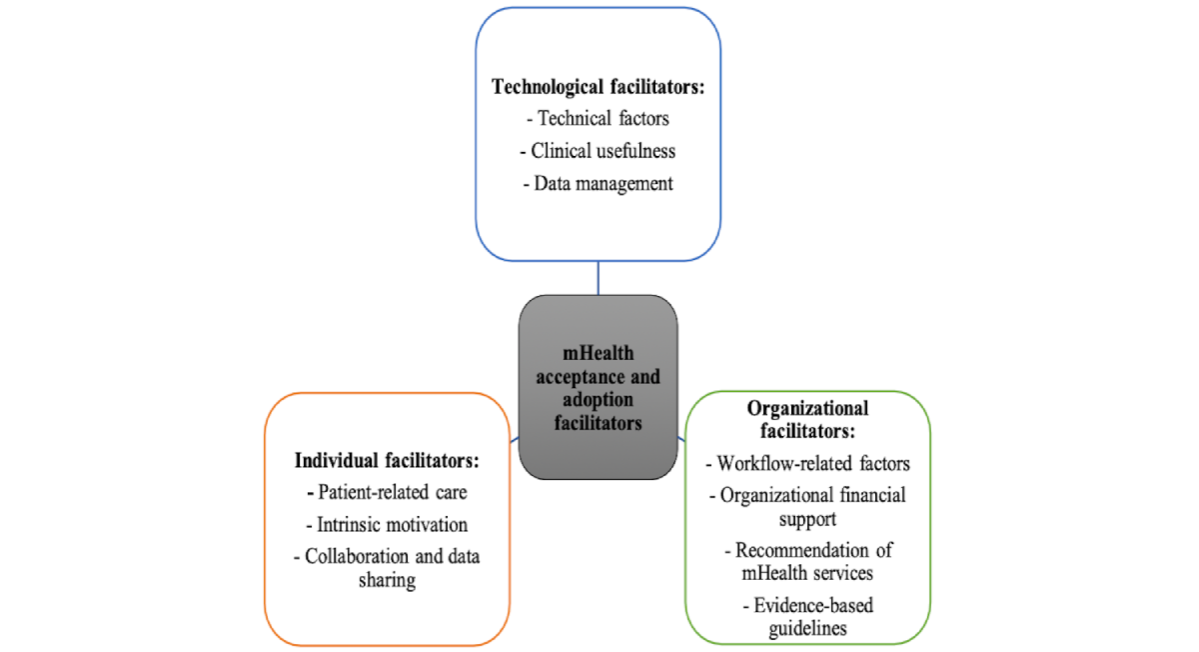
Themes of facilitators of mobile health (mHealth) acceptance and adoption by physicians.

All the included studies discussed the perceived facilitators of mHealth acceptance and adoption by health care providers (Table 3). Similar to the barriers, the facilitators were categorized into technological, individual, and organizational facilitators (Figure 3).

**Table 3:** Facilitators of the acceptance and adoption of mobile health (mHealth) technologies among physicians.

Study	Technological facilitators	Individual facilitators	Organizational facilitators
Aquino et al [54]	Evidence-based action prompts generated from patient data based on guidelines for patients at a high risk for preeclampsia Functionality: automatic data entry into telemonitoring systems	Perceived benefits: self-management tool for patients Effective display of patient data to facilitate trend detection and the visualization of patient health status	Facilitation of decision-making for clinicians by integrating evidence-based protocols and standards for patients at a high risk for preeclampsia
Artanian et al [28]	Functionality: ease of use of telemonitoring systems and their seamless integration into clinical practice and patient’s daily routine Clinical utility: access to daily data for providing accurate information about patient well-being	Engagement of eligible patients in telemonitoring	Availability of organizational resources: dedicated staff Advantageous over standard care owing to overcoming limitations in clinic space and the optimization of clinical resources Establishment of reimbursement models Adequate information on how to implement telemonitoring
Bhatt and Chakraborty [55]	Streamlined data handling for patient care management	Self-confidence or self-efficacy of physicians in handling technology requirements Performance expectancy Personal innovativeness	NR^a^
Dahlhausen et al [56]	Clinical utility: data and more accessible medical evidence Functionality: opportunities to navigate or test mHealth apps Additional information about mHealth platforms Compatibility of mHealth with existing infrastructures and workflows	Patient motivation or patient request to use mHealth tools	Recommendations by peers or medical associations Provision of provider reimbursement for mHealth-related medical services Extensive training with incentives (eg, certification for continuing medical education)
Fleddermann et al [57]	Integration of technology use into routine workflows Technological support for facilitating engagement	Significant levels of clinician engagement for supporting patient use of mHealth platforms, especially for supporting the management of challenges encountered by patients unable to access typical in-person treatment during isolation Collaboration with other staff using mHealth technologies	Recommendation by physicians for potential mHealth benefits Ongoing training
Jackson et al [58]	Functionality: patient-centered participatory design of customized functions and educational features, including data-tracking, motivational feedback, and bidirectional communication capabilities Clinical utility: potential to streamline clinical activities and resources Clinical integration (into routine prenatal care)	Provision for continued practical patient education to promote self-care management Clinician engagement with patient education	Integration of activities related to behavioral health changes into the patient’s daily routine
Li et al [59]	Functionality alert: function for the early detection of issues and timely interventions User-friendly and comes with an automatic data capture feature Access to data from multiple sources and integration of data with health records Demonstration of impact and evidence-based evaluation studies before implementation Compatibility with current practices of risk assessment and care for pregnant women with potential for multidisciplinary approach	Integrated tailored educational content and feedback for pregnant women based on conditions and risks Additional education and monitoring for pregnant women at high risk to improve data collection compliance and engagement	Recommendations by clinicians with indications for potential benefits Provision of ongoing education and training on using mHealth technologies
Mansour [60]	Simplicity, user-friendliness, and convenience of mHealth apps (eg, detection of COVID-19 symptoms, pulse oximeter, and COVID-19 health-monitoring apps) Access to COVID-19–related services and updated information Clinical utility: support for frequent health monitoring and preventive health care	Self-confidence in using technology Increased patient knowledge, improved patient engagement and medication adherence, and faster access to providers Communication and consultation with peers or colleagues and data sharing with other providers	Recommendation of mHealth use by physician
Wu et al [61]	Effort expectancy (ease and simplicity of mHealth)	Behavioral intention of physicians to use mHealth was significantly affected by intrinsic motivations (altruism and cognitive trust) High internet-based ratings affect sense of self-worth and contribute to positive participation in web-based health services	Integration of mHealth into the national health system Facilitating conditions, such as technical and human resource support, have a positive effect on mHealth adoption

^a^NR: not reported.

##### Technological Facilitators

The 3 main subthemes related to technological facilitators were technical factors, clinical usefulness, and data management. Technical factors were subdivided into access, functionality, and technical support domains, which were discussed in most of the reviewed studies (8/9, 89%) [28,54,56-61]. Specifically, of the 9 reviewed studies, 7 (78%) highlighted functionality and ease of use as important features for engaging providers [28,54,56,58-61]. For instance, the clinicians participating in these studies applauded the ease with which patients with diabetes can use mHealth systems to track blood sugar levels in real time and the advantage of direct feeds to providers [54]. Technological support was a critical facilitator of mHealth use [57]. Of the 9 studies, 2 (22%) identified access to mHealth services, such as data collection from multiple sources [59] and updated information [60], as facilitators of successful uptake by health care providers.

Among the 9 included studies, 5 (56%) discussed clinical utility and usefulness as factors that favor adoption [28,56,58-60]. Providers are more likely to use mHealth services when they perceive mHealth technologies as potentially streamlining patient management care and clinical resources; some examples are technologies that allow the monitoring of prescription changes and updating of medical charts or clinical notes [58]. Usefulness pertained primarily to the availability of accurate real-time information about patient well-being [28], additional support for current prenatal care practices [59], and frequent health monitoring [60]. Evidence-based evaluation studies and accessible evidence of the usefulness of mHealth platforms also potentially facilitate the adoption of mHealth technologies by health care providers [56,59]. Finally, facilitators that support adoption and sustained use were data management, including the integration of mHealth technologies into routine clinical practice and health records [28,56-59] and streamlined data handling for patient management [55].

##### Individual Facilitators

Individual facilitators were divided into the following 3 central subthemes: patient-related care, intrinsic motivation, and collaboration and data sharing. Facilitators related to patients were central in most of the reviewed studies (7/9, 78%). These included the perceptions (of physicians) that mHealth technologies have the potential to support self-managed care and provide real-time feedback [54,58], allow faster access to health care providers [60], integrate mHealth into patient routines with tailored content [59], improve patient engagement [58-60], and provide support to patients who are unable to access typical in-person clinical treatment given the isolation prompted by the COVID-19 pandemic [57]. In particular, physicians are predisposed to use mHealth services when their integration increases the efficiency of daily patient flow, data management, patient diagnosis, and other clinical activities [55,58]. During the pandemic, especially when clinic access was largely restricted, the promotion of mHealth as a patient self-care management tool was one of the key factors in physicians’ decision to adopt this innovation as a critical supportive tool in clinical care [54,57,58]. This decision is further supported by the effectiveness of mHealth in advancing multidisciplinary communication, as is the case, for example, with pregnancy care, for which access to data from multiple disciplines or sources is needed [59]. In addition, health care professionals with self-confidence, self-efficacy [55,60], altruism, and cognitive trust [61] in the reliability of technology are inclined to engage with and use mHealth platforms. These factors were rounded up through collaboration with peers or other users to share experiences and knowledge as well as data sharing with other providers [56,57,60].

##### Organizational Facilitators

Organizational facilitators were divided into the following 4 key subthemes: workflow-related factors, organizational financial support, recommendation of mHealth services, and evidence-based guidelines. Among the 9 included studies, 3 (33%) pinpointed workflow-related factors, such as the availability of support for streamlining clinical resources and activities and improvement of infrastructure for seamless workflow, as key facilitators [28,58,61]. In particular, organizational human resource support, such as the hiring of a dedicated coordinator to reduce physician workload [28] and address training needs [56,57,59,61], was highly advocated as a facilitator of mHealth uptake by physicians. Moreover, widespread adoption was found to be motivated by organizational financial support deployed via the establishment of reimbursement models [28] and the provision of financial incentives or reimbursement for mHealth services [56]. Effective implementation was also regarded as facilitated by the recommendation of mHealth services by trusted leaders, such as medical associations [56] or other physicians [57,59]. Other important facilitators of successful uptake included the integration of evidence-based standards and guidelines for telemonitoring into practice to facilitate clinical decision-making [54] and the integration of mHealth into the national health system [61]. None of the included studies reported specific facilitators regarding legal issues related to the security and privacy of patient data.

## Discussion

### Summary of the Main Findings

The COVID-19 pandemic has clearly been a catalyst of the wider acceptance and adoption of mHealth interventions worldwide, with studies frequently reporting benefits such as minimized risk of transmission, increased patient involvement, and reduced burden on hospitals and health care expenditure [9,62,63]. Nevertheless, the move toward mHealth apps as a model of care delivery during the pandemic has revealed several shortcomings in stimulating physicians’ uptake of such technologies. This review explored the factors influencing mHealth acceptance and adoption by physicians as the COVID-19 pandemic evolves. Factors related to the technological, individual, and organizational domains were identified.

### Critical Barriers to mHealth Acceptance and Adoption

Evidence suggests that a number of barriers have persisted since the prepandemic period [42,48,49]. This finding corresponds to the work of Zakerabasali et al [42], who reviewed evidence from 18 articles and identified 18 technical, individual, and health care system barriers. Similar to the findings in this review, the authors identified the lack of technical infrastructure, concerns about privacy issues, and the lack of workflow compatibility as barriers to mHealth adoption. Other principal barriers were limited technical literacy, preference for face-to-face interaction, financial factors, and health system policies [42]. Another prepandemic systematic review conducted in 2020 identified 55 barriers, including the lack of clinical training, the lack of technical support, the lack of compatibility with the existing workflow, and patient-related factors [48]. Consistent with the aforementioned studies, a systematic review conducted in 2016 identified 81 barriers, with emphasis placed on cost and time issues as well as difficulties in patient-professional interaction [49].

Although some of the perceived barriers that we found were similar to those identified in explorations carried out before the pandemic, we were able to identify other factors that are specific to acceptance and adoption during the pandemic. Examples include challenges accompanying the shift to hybrid care delivery to retain patients affected by the implementation of mHealth tools by physicians. The transition to internet-based treatment during the COVID-19 pandemic has disrupted services by dramatically reducing clinical caseloads, an issue that highlights patients’ preference for face-to-face appointments. Clinicians also lamented the considerable difficulty involved in assisting and guiding patients in downloading and signing up to an mHealth app [57]. As can be seen, the pandemic has highlighted the need to improve organizational readiness by making workflow adjustments to allow time for the introduction of mHealth tools to patients and the effective implementation of such innovations in practice. Another novel finding of this systematic review is that physicians perceive low competence in dealing with mHealth technologies as a result of insufficient knowledge and information regarding differentiating between mHealth platforms [56]. Collectively, these findings point to the importance of organizational support during *business as usual* periods to provide physicians with adequate education and training on the use of emerging mHealth tools.

Systematic reviews conducted before the pandemic differently emphasized barriers to mHealth adoption. Whereas cost issues and patient-professional interaction were reported as the most common barriers in an early systematic review [49], technical difficulties, particularly the lack of technical support, the lack of compatibility with the existing workflow, and patient-related challenges, were underscored as principal impediments in a more recent analysis [48]. In addition to technical and cost factors, privacy concerns were one of the most cited barriers in the examined studies [42]. To these lists, our study added limited financial support and technical and privacy issues as common barriers to uptake. However, in contrast to prepandemic reviews, this review identified patient-related factors, such as patient preference, engagement, and compliance, as the most frequently reported determinants of uptake during the pandemic. On these bases, we can conclude that the pandemic has shifted the focus from a technological perspective to a more patient-centered perspective in recognizing the main challenges to mHealth adoption and integration into practice.

### Leading Facilitators of mHealth Acceptance and Adoption

Some of the common facilitators of mHealth uptake evaluated in this study were consistent with those reported before the pandemic. These include perceived usefulness and ease of use, perceived patient-related benefits (eg, improved patient care, interprofessional collaborations, and data sharing), ongoing technical support and training, and financial support for technology implementation and integration with practice systems [48,49]. However, this review is distinct from prior research in terms of facilitators that are specific to the context of the pandemic.

The most prominent facilitators before the pandemic were those related to organizational workflow, such as infrastructure, training, resource allocation, perceived efficiency, improved reimbursement, and compatibility with workflow [48,49]. Against the backdrop of the pandemic, the central facilitators were the individual factors associated with the intrinsic motivation of physicians and patient-related matters. For instance, the behavioral intention of physicians to use mHealth apps was significantly influenced by self-efficacy [55], and intrinsic motivation was potentially strengthened by altruism and cognitive trust (perceived reliability) linked to competence in using mHealth platforms [61]. Recent studies confirmed that cognitive trust strongly influences the use of digital technologies, suggesting that it is essential to cultivate physicians’ trust in mHealth adoption through their sense of altruism [64] while their self-efficacy in the sustained intention to use mHealth platforms is elevated [65].

In our review, individual factors related to patient acceptance for greater engagement in and long-term commitment to using mHealth services were demonstrated to be critical to sustained uptake by physicians. High levels of physician engagement in promoting the benefits of mHealth apps for treatment [57] and clinician involvement with patient education [58] were also regarded as necessary for supporting patient access and the use of mHealth tools. This was especially important during periods of enforced isolation, as mHealth use fostered connections and supported the management of patients unable to access face-to-face treatment [57].

Furthermore, although addressing legal issues was one of the organizational factors that facilitated mHealth adoption before the pandemic [48], none of the reviewed studies discussed security and data protection. This deficiency can be attributed to the changes to regulations made by some countries during the global outbreak to provide further security guidance and support the more extensive use of telehealth [66]. In this situation, the attention of physicians could have been diverted from legal issues to concerns about their patients. Altogether, the available evidence highlights the importance of physicians’ intrinsic self-motivation in supporting a patient-centered approach. The focus should be directed to patient benefits as critical facilitators of successful acceptance and adoption in the context of the COVID-19 pandemic.

It is worth noting that there are varying factors influencing the acceptance and adoption of mHealth across limited-resource and high-resource countries. For instance, in limited-resource countries, Mansour [60] and Bhatt and Chakraborty [55] highlighted barriers, including the lack of language, technical skills, and training. By contrast, some studies in high-resource countries emphasized that mHealth apps were easy to use and integrated well into clinicians’ routines [28,58]. This variation can be attributed to the fact that health care systems in high-resource countries commonly have well-established training programs that integrate the latest medical advancements for health care professionals. By contrast, limited-resource countries may face challenges in providing sufficient training and education programs for health care professionals because of limited resources and funding [67-70]. Consequently, health care professionals in limited-resource countries may have limited opportunities for training and may not have the same skills and knowledge as their peers in high-resource countries.

Although our findings indicate that health care professionals have a generally positive attitude toward mHealth, there are variations in attitudes across various medical specialties [56,60]. For example, Dahlhausen et al [56] highlighted that neurologists have a mostly favorable perspective toward mHealth apps, whereas orthopedists and trauma surgeons hold somewhat less positive attitudes toward these apps. In line with our findings, a survey conducted by Zaslavsky et al [39] revealed differences in attitudes toward implementing mHealth apps across different medical specializations. Understanding these differences is crucial for customizing strategies to promote the adoption of mHealth among various medical specialties.

### Limitations and Recommendations for Future Research

Although this review contributes to the understanding of the factors influencing the acceptance and adoption of mHealth technologies among physicians, some limitations must be acknowledged. Most studies (6/9, 67%) were conducted in developed countries (eg, the United States, Canada, and Germany) [28,54,56,57], which means that our understanding of the factors influencing the acceptance and adoption of mHealth among physicians in developing countries is limited. Moreover, more than half (5/9, 56%) of the studies [28,54,57-59] used qualitative methods, such as semistructured interviews and focus group discussions, to gather data. Therefore, generalizing the results of this review may be challenging. In addition, this review might not have incorporated relevant papers that were not listed in the databases that were searched and that were published in a language other than English, which would have helped identify more factors that influence the acceptance and adoption of mHealth among physicians.

We provide several recommendations for future research. Identifying the factors that affect the acceptance and adoption of technologies such as mHealth is an ongoing process [57]. Hence, there is a need for more extensive research on these behaviors of physicians, especially in limited-resource countries. Research in limited-resource countries is necessary to understand whether there are different opportunities and constraints. In addition, robust methodologies, such as mixed methods approaches, are required to uncover the factors influencing acceptance and adoption. Mixed methods research can overcome the disadvantages associated with quantitative or qualitative approaches, thereby enriching the findings. For example, some researchers claim that quantitative exploration insufficiently advances the understanding of contexts or areas in which people live, as the voices of participants are not directly heard [71]. Qualitative studies might be considered deficient because of a researcher’s subjective interpretations, the bias that results from these, and the difficulty in generalizing findings [71]. Finally, the identified factors could help policy makers make decisions aimed at implementing mHealth successfully. These factors may facilitate physicians’ acceptance and adoption of mHealth technologies.

### Conclusions

The pandemic has highlighted and expanded the avenues in which mHealth can aid clinical decision-making and improve the quality of care. This review summarized the evidence on the factors influencing mHealth acceptance and adoption by physicians during the COVID-19 pandemic. The main findings of this review highlighted the importance of addressing organizational readiness to support physicians with adequate resources, shifting the focus from technological to patient-centered factors, and the seamless integration of mHealth into routine practice during and beyond the pandemic.

## Appendix

Multimedia Appendix 1 PRISMA (Preferred Reporting Items for Systematic Reviews and Meta-Analyses) 2020 checklist.

Multimedia Appendix 2 Search strategy.

Multimedia Appendix 3 Risk-of-bias assessment of the included studies.

## Abbreviations

MeSH: Medical Subject Headings
mHealth: mobile health
PICO: population, intervention, comparator, and outcome
PRISMA: Preferred Reporting Items for Systematic Reviews and Meta-Analyses
QuADS: Quality Assessment with Diverse Studies
WHO: World Health Organization
