# Supplementary material for: Factors Influencing the Acceptance and Adoption of Mobile Health Apps by Physicians During the COVID-19 Pandemic: Systematic Review
Source: JMIR Mhealth Uhealth. 2023 Nov 8;11:e50419. doi: 10.2196/50419 (PMC10666016; doi:10.2196/50419)
Supplement: Multimedia Appendix 2 [file mhealth_v11i1e50419_app2.docx]

### Multimedia Appendix 2

Search strategy

Although the search strategy was the same for each database, modifications were made to consider the different interfaces.

Example of a search strategy:

Medline

1 mhealth.mp.

2 m-health.mp.

3 mobile health.mp.

4 1 or 2 or 3

5 physician.mp. or Physicians/

6 doctor.mp.

7 practitioner.mp. or General Practitioners/

8 clinician.mp.

9 5 or 6 or 7 or 8

10 Adoption.mp. or Adoption/

11 Adopt*.mp.

12 Accept*.mp.

13 Barrier*.mp.

14 Attitude/ or Attitude*.mp.

15 10 or 11 or 12 or 13 or 14

16 4 and 9 and 15

17 limit 16 to (english language and yr="2020 - 2022" and covid-19)

Scopus (Elsevier)

(TITLE-ABS-KEY(m-Health OR mhealth OR "mobile health" OR "Mobile app") AND TITLE-ABS-KEY(adopt* OR attitude* OR accept* OR Barrier*) AND TITLE-ABS-KEY(Physician* OR Doctor* OR Practitioner* OR Clinician*)) AND ( LIMIT-TO ( DOCTYE,"ar" ) ) AND ( LIMIT-TO ( SRCTYPE,"j" ) ) AND ( LIMIT-TO ( EXACTKEYWRD,"MHealth" ) OR LIMIT-TO ( EXACTKEYWORD,"Mobile Health" ) OR LIMIT-TO ( EXACTKEYWORD,"Mobile Application" ) OR LIMIT-TO ( EXACTKEYWORD,"Digital Health" ) OR LIMIT-TO ( EXACTKEYWORD,"COVID-19" ) OR LIMIT-TO ( EXACTKEYWORD,"Mhealth" ) OR LIMIT-TO ( EXACTKEYWORD,"M-health" ) ) AND ( LIMIT-TO ( PUBYEAR,2022) OR LIMIT-TO ( PUBYEAR,2021) OR LIMIT-TO ( PUBYEAR,2020) ) AND ( LIMIT-TO ( LANGUAGE,"English" ) )

Embase

1 mhealth.mp.

2 m-health.mp.

3 mobile application/ or mobile health.mp. or mobile health application/

4 1 or 2 or 3

5 physician/ or physician.mp.

6 Doctor*.mp.

7 practitioner*.mp. or general practitioner/ or health practitioner/

8 clinician/ or clinician*.mp.

9 5 or 6 or 7 or 8

10 adopt*.mp. or adoption/

11 Accept*.mp.

12 Barrier*.mp.

13 health personnel attitude/ or attitude*.mp.

14 10 or 11 or 12 or 13

15 4 and 9 and 14

16 limit 15 to (english language and yr="2020 - 2022" and covid-19)

ProQuest

((ab(m-Health OR mhealth OR "mobile health" OR "Mobile app") AND ab(adopt* OR attitude* OR accept* OR Barrier*) AND ab(Physician* OR Doctor* OR Practitioner* OR Clinician*) AND stype.exact("Scholarly Journals") AND la.exact("English")) NOT (subt.exact("patients" OR "teenagers" OR "nurses" OR "older people") AND PEER(yes))) AND pd(20200301-20221231)
