# Supplementary material for: Factors Influencing the Acceptance and Adoption of Mobile Health Apps by Physicians During the COVID-19 Pandemic: Systematic Review
Source: JMIR Mhealth Uhealth. 2023 Nov 8;11:e50419. doi: 10.2196/50419 (PMC10666016; doi:10.2196/50419)
Supplement: Multimedia Appendix 3 [file mhealth_v11i1e50419_app3.docx]

## Multimedia Appendix 3

Risk-of-bias assessment of the included studies

| No | **Criteria \ Score** | Aquino et al. ^[^54^]^ | Artanian et al. ^[^28^]^ | Bhatt and Chakraborty ^[^55^]^ | Dahlhausen et al. ^[^56^]^ | Fleddermann et al. ^[^57^]^ | Jackson et al. ^[^58^]^ | Li et al. ^[^59^]^ | Mansour ^[^60^]^ | Wu et al. ^[^61^]^ |
| --- | --- | --- | --- | --- | --- | --- | --- | --- | --- | --- |
| 1 | Theoretical or conceptual underpinning to the research | 0 | 1 | 3 | 0 | 3 | 2 | 0 | 0 | 3 |
| 2 | Statement of research aim/s | 3 | 3 | 3 | 3 | 2 | 3 | 3 | 1 | 3 |
| 3 | Clear description of research setting and target population | 3 | 3 | 1 | 3 | 3 | 3 | 3 | 1 | 1 |
| 4 | The study design is appropriate to address the stated research aim/s | 3 | 3 | 2 | 3 | 3 | 3 | 3 | 3 | 3 |
| 5 | Appropriate sampling to address the research aim/s | 3 | 3 | 2 | 3 | 2 | 2 | 1 | 1 | 2 |
| 6 | Rationale for choice of data collection tool(s) | 0 | 3 | 1 | 2 | 0 | 3 | 0 | 2 | 2 |
| 7 | The format and content of data collection tool is appropriate to address the stated research aim/s | 3 | 3 | 2 | 3 | 3 | 3 | 3 | 3 | 3 |
| 8 | Description of data collection procedure | 3 | 3 | 1 | 3 | 3 | 2 | 3 | 1 | 1 |
| 9 | Recruitment data provided | 1 | 3 | 0 | 2 | 3 | 3 | 2 | 2 | 3 |
| 10 | Justification for analytic method selected | 0 | 3 | 2 | 3 | 0 | 0 | 0 | 1 | 2 |
| 11 | The method of analysis was appropriate to answer the research aim/s | 3 | 3 | 3 | 3 | 3 | 3 | 3 | 3 | 3 |
| 12 | Evidence that the research stakeholders have been considered in research design or conduct | 0 | 0 | 0 | 2 | 0 | 0 | 0 | 1 | 0 |
| 13 | Strengths and limitations critically discussed | 1 | 3 | 0 | 1 | 2 | 3 | 2 | 1 | 2 |
|  | **Total** | 23 | 34 | 20 | 31 | 27 | 30 | 23 | 20 | 28 |
|  | **Maximum score possible** | 39 | 39 | 39 | 39 | 39 | 39 | 39 | 39 | 39 |
|  | **Score as Percentage (%)** | 59% | 87% | 51% | 79% | 69% | 77% | 59% | 51% | 72% |
